# Portable visual and electrochemical detection of hydrogen peroxide release from living cells based on dual-functional Pt-Ni hydrogels

**DOI:** 10.1038/s41378-023-00623-y

**Published:** 2023-11-29

**Authors:** Guanglei Li, Yao Chen, Fei Liu, Wenhua Bi, Chenxin Wang, Danfeng Lu, Dan Wen

**Affiliations:** 1 State Key Laboratory of Solidification Processing, School of Materials Science and Engineering, Northwestern Polytechnical University (NPU) and Shaanxi Joint Laboratory of Graphene, Xi’an, 710072 P. R. China; 2 Interdisciplinary Research Center of Biology & Catalysis, School of Life Sciences, NPU, Xi’an, 710072 P. R. China; 3 Faculty of Printing, Packaging Engineering, and Digital Media Technology, Xi’an University of Technology, Xi’an, 710048 P. R. China

**Keywords:** Nanoscience and technology, Chemistry, Engineering

## Abstract

It is important to monitor the intra-/extracellular concentration of hydrogen peroxide (H_2_O_2_) in biological processes. However, miniaturized devices that enable portable and accurate H_2_O_2_ measurement are still in their infancy because of the difficulty of developing facile sensing strategies and highly integrated sensing devices. In this work, portable H_2_O_2_ sensors based on Pt-Ni hydrogels with excellent peroxidase-like and electrocatalytic activities are demonstrated. Thus, simple and sensitive H_2_O_2_ sensing is achieved through both colorimetric and electrochemical strategies. The as-fabricated H_2_O_2_ sensing chips exhibit favorable performance, with low detection limits (0.030 μM & 0.15 μM), wide linearity ranges (0.10 μM–10.0 mM & 0.50 μM–5.0 mM), outstanding long-term stability (up to 60 days), and excellent selectivity. With the aid of an M5stack development board, portable visual and electrochemical H_2_O_2_ sensors are successfully constructed without complicated and expensive equipment or professional operators. When applied to the detection of H_2_O_2_ released from HeLa cells, the results obtained by the developed sensors are in good agreement with those from an ultraviolet‒visible spectrophotometer (UV‒vis) (1.97 μM vs. 2.08 μM) and electrochemical station (1.77 μM vs. 1.84 μM).

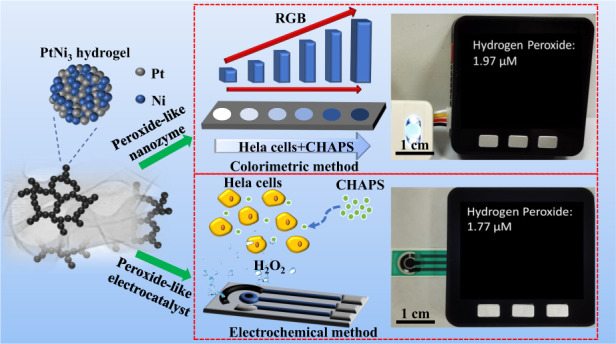

## Introduction

H_2_O_2_ is one of the most important metabolic products and plays a key role in the proliferation, differentiation, and migration of cells under physiological conditions^1,2^. However, excessive levels of H_2_O_2_ may be associated with various pathological conditions, such as cancer, Alzheimer’s disease and Parkinson’s disease^3–5^, which makes the accurate measurement of H_2_O_2_ urgent and important for preventing, diagnosing and treating these diseases. Among numerous analytical strategies for H_2_O_2_ determination, colorimetric and electrochemical approaches are considered the most powerful and versatile approaches due to their advantages of high sensitivity, simple operation and low cost^6–8^. Nevertheless, highly miniaturized and cost-effective H_2_O_2_ sensors that do not rely on large equipment and professional operators have rarely been explored. This is mainly due to the difficulties of developing facile sensing strategies and the efficient integration of both sensing chips and signal processing into sensing platforms^9^. In addition, typical H_2_O_2_ sensors are generally based on natural enzymes (e.g., horseradish peroxidase (HRP)), which are relatively fragile and expensive^10,11^, thus hindering their widespread application. Therefore, it is highly desirable to develop sensing materials with excellent catalytic activities to bypass the limitations imposed by natural enzymes and to fabricate H_2_O_2_ sensing devices that can meet the need for immediate, portable, and sensitive H_2_O_2_ detection.

Nanomaterials with properties such as mimicking enzymatic activities or electrocatalytic functions have attracted growing interest in recent years^12–14^. With the accelerated development of material science and technology, many nanozymes or electrocatalytic nanomaterials to replace natural enzymes owing to the easier large-scale production, lower cost, more tunable catalytic activities and higher stability of the nanomaterials^15–17^. Metal aero-/hydrogels derived from nanoparticles are an emerging type of self-supported, three-dimensional (3D) porous nanomaterials with excellent catalytic activity. They have gained great attention and displayed unprecedented potential in the research areas of electrocatalysis, nanozymes and biosensing^18–20^. For instance, Zhu et al. proposed a dopamine-induced Au hydrogel that exhibited high glucose oxidase-like and peroxidase-like activities, offering a new approach to designing biosensors with metal gel-based nanozymes^21^. The fascinating porous structures and large surface areas of the metal gels can greatly improve the diffusion of the substrate and provide a large number of active sites, which thus substantially enhances the sensitivity^22–24^. The 3D interconnected networks of the metallic skeleton guarantee high numbers of electron transfer pathways for catalytic reactions^25^. In addition, improved retention of the catalytic activity can significantly increase the long-term stability of biosensing compared to that of natural enzymes^26^. Therefore, metal gel sensing materials with these outstanding features will open up whole new vistas for the construction of sensing devices^27,28^.

Herein, we developed portable H_2_O_2_ sensors based on Pt-Ni hydrogels composed of alloyed nanowires and Ni(OH)_2_ nanosheets, which have demonstrated unexpected peroxidase-like and electrocatalytic properties toward H_2_O_2_. The optimized PtNi_3_ hydrogel-based H_2_O_2_ sensing platforms displayed remarkable performance in both colorimetric and electrochemical methods with wide linearity ranges, low limits of detection (LODs), robust long-term stability and good selectivity against common interferences. Together with an M5stack development board, a portable visual H_2_O_2_ sensor with an integrated PtNi_3_ hydrogel-based colorimetric test paper and a portable electrochemical H_2_O_2_ sensor with an integrated PtNi_3_-modified screen-printing electrode (SPE) were successfully constructed. Finally, accurate quantitative analysis of H_2_O_2_ released from HeLa cells was achieved, revealing the practicability of the proposed biosensors. This work not only demonstrates the versatility of metal hydrogels but also provides a new strategy for high-performance and low-cost sensor design.

## Results and discussion

### Microstructures of the Pt-Ni hydrogels

**Fig. 1:**
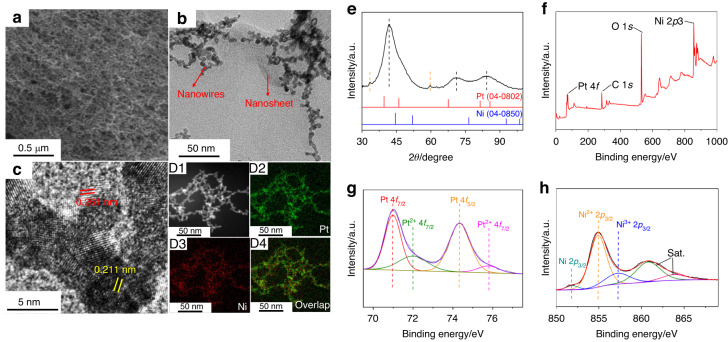
Characterization of the PtNi3 hydrogel. **a** SEM, **b** TEM, **c** HRTEM, **d** elemental mapping images, **e** XRD pattern, **f** XPS wide scan, and high-resolution (**g**) Pt 4 f and (**h**) Ni 2p spectra of the PtNi_3_ hydrogel

The Pt-Ni hydrogels were prepared via a fast and simple coreduction of a mixed metal salt solution by sodium borohydride (NaBH_4_). Scanning and transmission electron microscopy (SEM and TEM) were first carried out to evaluate the morphology and microstructure of the representative PtNi_3_ hydrogel (see Fig. 1a–d). It displayed a highly porous dual gel structure composed of interfused nanowire networks and crumpled nanosheets (Fig. 1a, b), which provided a large specific surface area and ensured high sensitivity for biosensing. The interplanar spacing value measured from the nanowire was 0.211 nm in the high-resolution TEM image (Fig. 1c), which could be assigned to the (111) facet of Pt. This value was smaller than that of metallic Pt, indicating the possible formation of a Pt-Ni alloy. Moreover, the interplanar spacing of the nanosheets was 0.261 nm, indexing to the (100) facets of Ni(OH)_2_. The diffraction peaks at 41.83°, 71.16°, and 84.26° were located between those expected for metallic Pt (PDF 04-0802) and Ni (PDF 04-0850), further demonstrating the lattice contraction of Pt and the formation of a Pt-Ni alloy in the PtNi_3_ hydrogel (Fig. 1e). Moreover, the diffraction peaks at 33.48° and 59.70° corresponded to the (100) and (003) reflection planes of Ni(OH)_2_, respectively. Both the TEM and X-ray diffraction (XRD) results revealed the dual-structure gels of the Pt-Ni alloyed nanowires and Ni(OH)_2_ nanosheets. In addition, the high-resolution X-ray photoelectron spectroscopy (XPS) patterns of Pt 4 f and Ni 2p showed metallic and oxidative states, respectively, with electron transfer from Ni to Pt (Fig. 1f–h).

Pt-Ni hydrogels with different Pt/Ni atomic ratios (e.g., PtNi and PtNi_5_ hydrogels) could be obtained using this standard synthetic approach by tuning the amounts of metal precursors and NaBH_4_ (see details in the Experimental Section and Fig. S1). Additionally, pure Pt and Ni hydrogels were synthesized for comparison by reducing only the Pt or Ni precursor. From the SEM and TEM images of these hydrogels, wrinkled nanosheets were almost invisible in the pure Pt and PtNi hydrogels and clearly observed in the PtNi_5_ hydrogel, indicating that the amount of Ni(OH)_2_ increased as the proportion of Ni increased.

### Peroxidase-like activity and H_2_O_2_ colorimetric sensing

**Fig. 2:**
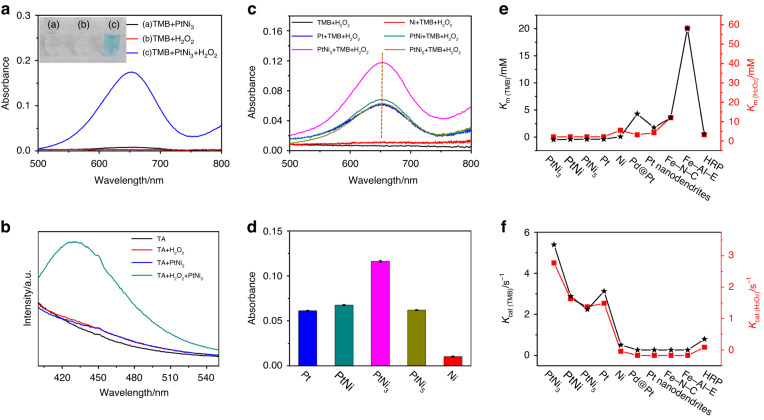
Investigation of the peroxidase-like characteristics of the PtNi_x_ hydrogels. **a** Typical absorption spectra of the PtNi_3_ hydrogel in 0.50 mM TMB solution; **b** the measurement of •OH by the fluorescence absorption spectra in 0.50 mM TA solution. The concentrations of H_2_O_2_ and the PtNi_3_ hydrogel were 0.50 mM and 0.050 μg mL^−1^, respectively; **c** typical absorption spectra of the Pt, Ni, and PtNi_x_ hydrogels in 0.10 M phosphate-buffered saline (PBS) (pH 5.0) with 0.50 mM TMB and 0.20 mM H_2_O_2_; **d** the absorbance peaks of the Pt, Ni, and PtNi_x_ hydrogels at 652 nm; (**e**, **f**) comparisons of the *K*_*m*_ and *K*_*cat*_ of the PtNi_x_, pure Pt, Ni hydrogels and other artificial/natural enzymes

H_2_O_2_ is an important biomarker in living cells and an intermediate product in the oxidation of many biomolecules (e.g., glucose, lactate and alcohol), thus making H_2_O_2_ measurement a prevalent application in analytics and diagnostics^29–31^. It is well known that Pt-based nanomaterials show peroxidase-like activity and have great potential in colorimetric H_2_O_2_ sensing^32^. First, the peroxidase-like characteristics of the PtNi_3_ hydrogel were investigated through a 3,3,5,5-tetramethylbenzidine (TMB)-induced chromogenic reaction and UV‒vis absorption spectra. As displayed in Fig. 2a, neither the PtNi_3_ hydrogel nor the H_2_O_2_ solution and TMB yielded a color change or obvious absorption peak. In contrast, the mixed solution of TMB and PtNi_3_ hydrogel turned from transparent to blue in the presence of H_2_O_2_. The time-dependent absorption spectrum in Fig. S2 shows that the curve reached a steady state within 3 min, indicating a rapid response time. The characteristic peak at 652.0 nm was assigned to the oxidation of TMB (ox-TMB), demonstrating the peroxidase-like activity of the PtNi_3_ hydrogel. In addition, a terephthalic acid (TA)-induced chromogenic reaction was carried out to explore the catalytic mechanism. A characteristic peak at 430 nm appeared only when there were both H_2_O_2_ and PtNi_3_ hydrogels in the solution (Fig. 2b). This phenomenon was due to the fluorescent product of the reaction of TA with hydroxyl radical (•OH)^33^. Therefore, the nature of the catalytic behavior of the PtNi_3_ hydrogel can be attributed to the generation of •OH^34^.

For comparison, we tested the peroxidase-like activity of the Pt, Ni, PtNi and PtNi_5_ hydrogels. As seen in Fig. 2c, d, all the Pt-based hydrogels exhibited significant absorbances (Pt: 0.062, PtNi: 0.068, PtNi_3_: 0.117, PtNi_5_: 0.063) in the presence of 0.20 mM H_2_O_2_, while only a slight signal was observed in the pure Ni hydrogel. The steady-state kinetic assay of the PtNi_3_ hydrogel was performed by varying the TMB or H_2_O_2_ concentrations under the same conditions. Control experiments were also carried out for the PtNi, PtNi_5_, pure Pt and Ni hydrogels. Both TMB and H_2_O_2_ followed the standard Michaelis‒Menten model well for these hydrogels (Figs. S3–S7). The corresponding Michaelis constant (*K*_*m*_), maximum initial velocity (*V*_*max*_) and catalytic constant (*K*_*cat*_) of the PtNi_x_, pure Pt and Ni hydrogels were calculated according to the Lineweaver‒Burk curves and are shown in Table S1 and Fig. 2e, f^34,35^. The *K*_*m*_ values of the Pt-based hydrogels for both H_2_O_2_ and TMB were much lower than that of HRP, suggesting their higher affinity to these two substrates^36^. The higher *K*_*cat*_ of these hydrogels with respect to HRP indicated higher catalytic activity per unit concentration^37^. Most importantly, the PtNi_3_ hydrogel displayed the highest affinity and catalytic activity (*K*_*m (TMB)*_: 0.031 mM, *K*_*m (H2O2)*_: 0.67 mM, *V*_*max (TMB)*_: 6.08 × 10^−8^ M s^−1^, *V*_*max (H2O2)*_: 4.52 × 10^−8^ M s^−1^) compared to the natural and artificial enzymes (Table S1). This can be ascribed to the following three factors. (1) The highly porous structures and large surface area of the PtNi_3_ hydrogel provide excellent mass transfer and rapid electron transfer between the hydrogels and the substrates^25^. (2) Considering the low catalytic activity of the Ni hydrogel, we can infer that the high peroxidase-like activity of the PtNi_3_ hydrogel was promoted mainly due to the synergistic effect between Pt and Ni in the alloyed Pt-Ni nanowires^38^. (3) Moreover, the generated Ni(OH)_2_ nanosheets can significantly enhance the affinity of the PtNi_3_ hydrogel to the substrates by increasing the specific surface area and accelerating the desorption of the •OH adsorbed on the PtNi alloy, thus improving its catalytic performance^39–41^. However, excessive Ni(OH)_2_ on the surface of the hydrogel may restrict the contact between the substrates and the alloyed Pt-Ni nanowires, reducing the catalytic performance (e.g., PtNi_5_ hydrogel) and resulting in a volcano-type trend with increasing Ni content, as shown in Fig. 2d.

Taking into account the excellent peroxidase-like property, the PtNi_3_ hydrogel shows great potential for constructing a colorimetric H_2_O_2_ sensor. The working temperature and pH value were optimized to maximize the sensing performance. Similar to the natural enzymes, the catalytic activity of the PtNi_3_ hydrogel exhibited temperature-dependent (Fig. S8A, B) and pH-dependent (Fig. S8C, D) behavior. Therefore, the optimal conditions of 25 °C and pH = 5 were selected for the subsequent sensing experiments.

**Fig. 3:**
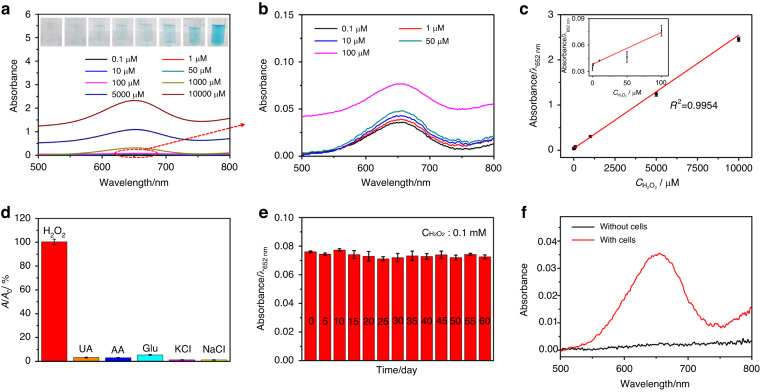
Analytical performance of the PtNi_3_ hydrogel in H_2_O_2_ colorimetric sensing. **a**, **b** The absorption spectra of the PtNi_3_ hydrogel in the presence of different concentrations of H_2_O_2_ in 0.10 M PBS (pH 5.0) with 0.50 mM TMB at 25 °C, inset (**a**) is the optical images of the TMB solution with different concentrations of H_2_O_2_; (**c**) the corresponding calibration curve of H_2_O_2_ versus the absorbance at 652.0 nm, inset is the calibration curve in the range of 0.10–100.0 μM; (**d**) the selectivity and (**e**) long-term stability of the PtNi_3_ hydrogel for H_2_O_2_ sensing; (**f**) the detection of H_2_O_2_ in the HeLa cells

We then systematically evaluated the analytical performances in H_2_O_2_ colorimetric sensing based on the PtNi_3_ hydrogel. Figure 3a illustrates the UV‒vis spectra and corresponding optical images of the TMB solution with different concentrations of H_2_O_2_. As shown in Fig. 3a–c, the absorbance of ox-TMB at 652.0 nm increased with increasing H_2_O_2_ concentration from 0.10 μM to 10.0 mM and revealed a wide linearity range. The LOD was calculated to be 0.030 μM (S/N = 3). Furthermore, the tiny relative standard deviation (RSD) of nine repeated measurements (less than 1.50%) indicated high repeatability of the H_2_O_2_ measurement. In addition, the selectivity of the PtNi_3_ hydrogel-based colorimetric sensing was evaluated. The results in Fig. 3d show almost no significant signal changes in the absorbance with respect to 0.10 mM H_2_O_2_ after the addition of 0.20 mM uric acid (UA) (3.24%), 0.20 mM ascorbic acid (AA) (3.06%), 0.20 mM glucose (5.38%), 0.20 mM potassium chloride (KCl) (1.32%) and 0.20 mM sodium chloride (NaCl) (1.24%), demonstrating good selectivity. Moreover, the long-term stability of the PtNi_3_ hydrogel for H_2_O_2_ measurement was studied. The PtNi_3_ hydrogel from one batch was used to measure 0.10 mM H_2_O_2_ every 5 days. It can be seen from Fig. 3e that the catalytic activity was well maintained even after 60 days (RSD = 2.30%), indicating the outstanding long-term stability of the PtNi_3_ hydrogel-based sensing platform. These sensing performances were better than those of most reported colorimetric H_2_O_2_ sensors (see Table S2). Finally, we measured the released H_2_O_2_ from the 3-[(3-cholanidopropyl) dimethylammonio]-1-pro panesulfonate (CHAPS)-stimulated HeLa cells. From Fig. 3f, significant absorbance was observed after the addition of 0.50 μM CHAPS to the solution with HeLa cells (3.60 × 10^5^ cells mL^−1^), while there was no change in absorbance in the same solution without HeLa cells. The H_2_O_2_ concentration was calculated to be 2.08 μM, confirming the feasibility of developing an accurate and sensitive H_2_O_2_ sensor.

### Electrocatalytic activity and H_2_O_2_ electrochemical sensing

**Fig. 4:**
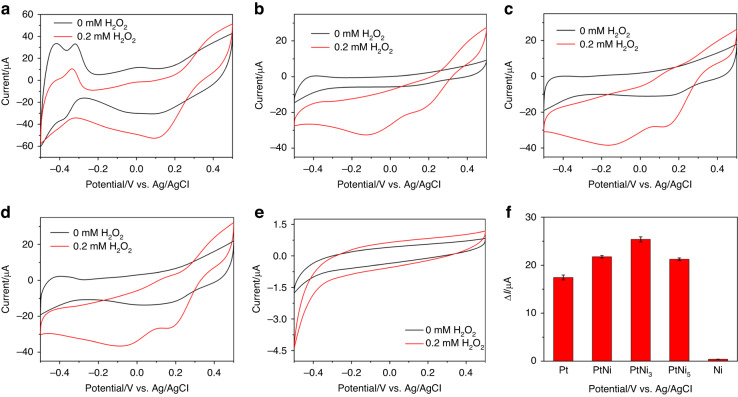
Investigation of the electrocatalytic activities of the PtNi_x_ hydrogels. CVs of the (**a**) Pt/GCE, (**b**) PtNi/GCE, (**c**) PtNi_3_/GCE, (**d**) PtNi_5_/GCE, and (**e**) Ni/GCE for 0.20 mM H_2_O_2_ in 0.10 M PBS (pH 7.40) at a scan rate of 50 mV s^−1^; the GCEs were modified with 3.0 µg of Pt, PtNi, PtNi_3_, PtNi_5_ and Ni hydrogels; (**f**) the corresponding current responses of 0.20 mM H_2_O_2_ at the above hydrogel-modified electrodes at −0.30 V

We also investigated the electrochemical characteristics of the Pt-Ni hydrogels in catalyzing H_2_O_2_. Figure 4 depicts the cyclic voltammograms (CVs) of the pure Pt, PtNi, PtNi_3_, PtNi_5_ and pure Ni hydrogel-modified electrodes in 0.10 M PBS (pH 7.40) containing 0.20 mM H_2_O_2_. The reduction currents of the Pt, PtNi, PtNi_3_, and PtNi_5_ hydrogels all clearly increased with the addition of H_2_O_2_, while the pure Ni hydrogel showed almost no response, demonstrating the high activities of the Pt-based hydrogels in the electrocatalytic reaction of H_2_O_2_. Among these hydrogels, the PtNi_3_ hydrogel exhibited the highest current response at a potential of −0.3 V, that is, 17.45 μA (Pt), 21.77 μA (PtNi), 25.40 μA (PtNi_3_), 21.24 μA (PtNi_5_), and 0.40 μA (Ni) (see Fig. 4f). This phenomenon can also be ascribed to the promotion of catalysis by the synergistic effect between Pt and Ni in the alloyed nanowires as well as the higher affinity provided by moderate Ni(OH)_2_ nanosheets^40,41^, which has been discussed before. Thus, the PtNi_3_ hydrogel was chosen for the subsequent experiments.

**Fig. 5:**
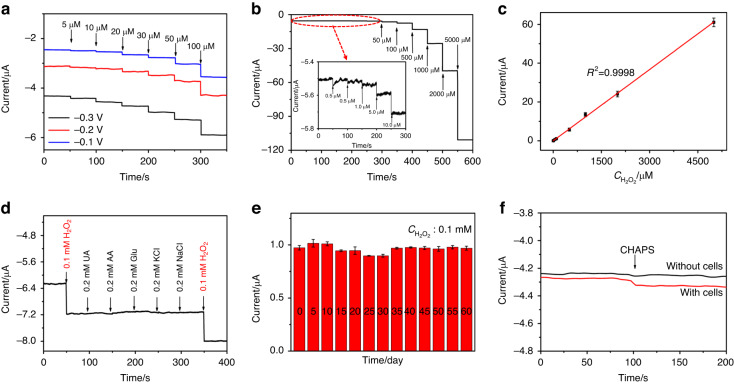
Analytical performance of the PtNi_3_ hydrogel in H_2_O_2_ electrochemical sensing. **a** The i-t curves of PtNi_3_/GCE at bias voltages of −0.10 V, −0.20 V and −0.30 V, respectively; **b** the i-t curve recorded at PtNi_3_/GCE with the successive addition of H_2_O_2_ under a bias voltage of −0.30 V and (**c**) the corresponding calibration curve; (**d**) the anti-interference experiment with 0.10 mM H_2_O_2_ and 0.20 mM interferences; (**e**) the current response of 0.10 mM H_2_O_2_ at PtNi_3_/GCE for 60 days; (**f**) the i-t curves of PtNi_3_/GCE responding to the H_2_O_2_ released from HeLa cells upon CHAPS stimulation

Then, the analytical performance of the PtNi_3_ hydrogel-based electrode for the electrochemical detection of H_2_O_2_ was evaluated via the amperometric i-t curves (i-t curves). Due to the wide catalytic potential window of H_2_O_2_ for the PtNi_3_ hydrogel, −0.30 V was selected as the optimized bias voltage due to the highest current response (see Fig. 5a) and the ability to avoid coexisting interferences. The results shown in Fig. 5b revealed that the current response increased rapidly with the concentration of H_2_O_2_. A high sensitivity of 172.82 μA mM^−1^ cm^−2^ was estimated within a wide linearity range of 0.50 µM–5.0 mM with an LOD of 0.15 µM (S/N = 3) according to the calibration curve given in Fig. 5c. Moreover, excellent selectivity was observed, as shown in Fig. 5d, as the common interfering substances (0.20 mM UA, 0.20 mM AA, 0.20 mM Glu, 0.20 mM KCl, 0.20 mM NaCl) had no significant current response with respect to 0.10 mM H_2_O_2_. Furthermore, we investigated the influence of pH on the electrocatalytic activity of the PtNi_3_ hydrogel. The results shown in Fig. S9 indicated that the electrocatalytic performance of the hydrogel was also pH dependent. The catalyst performed well in the weakly acidic environment but poorly in the strongly acidic or alkaline conditions. This is because strong acid will corrode the Ni(OH)_2_ nanosheets, while alkaline conditions will cause the decomposition of H_2_O_2_. Additionally, the long-term stability of the PtNi_3_ hydrogel in catalyzing H_2_O_2_ was also explored, and the result is given in Fig. 5e. The PtNi_3_ hydrogel displayed consistent current responses in the 60-day measurement (RSD = 3.48%), demonstrating its long-term stability. This outstanding stability could be explained by the following two reasons: (1) the PtNi_3_ hydrogel applied as the sensing material avoided the problem of activity attenuation of the natural enzymes, thus enhancing the chemical stability; (2) the physical stability of the hydrogel was guaranteed by the dual structure of the interfused nanowires and nanosheets, further promoting the long-term stability.

The excellent sensing performance endows the PtNi_3_ hydrogel with great application potential in complex biological systems. Therefore, we finally measured the H_2_O_2_ secreted from HeLa cells stimulated by CHAPS. In the presence of HeLa cells (3.60 × 10^5^ cells mL^−1^), an obvious current response was observed as soon as CHAPS (0.50 μM) was added (red line in Fig. 5f). The concentration of H_2_O_2_ was calculated to be 1.84 μM according to the calibration curve in Fig. 5c. In contrast, there was no obvious current change under the same experimental conditions in the absence of HeLa cells (black line in Fig. 5f), confirming that the measured H_2_O_2_ was generated from the cells. These results verified the further applicability of the PtNi_3_ hydrogel in sensing living cells.

### Portable visual and electrochemical H_2_O_2_ sensors

**Fig. 6:**
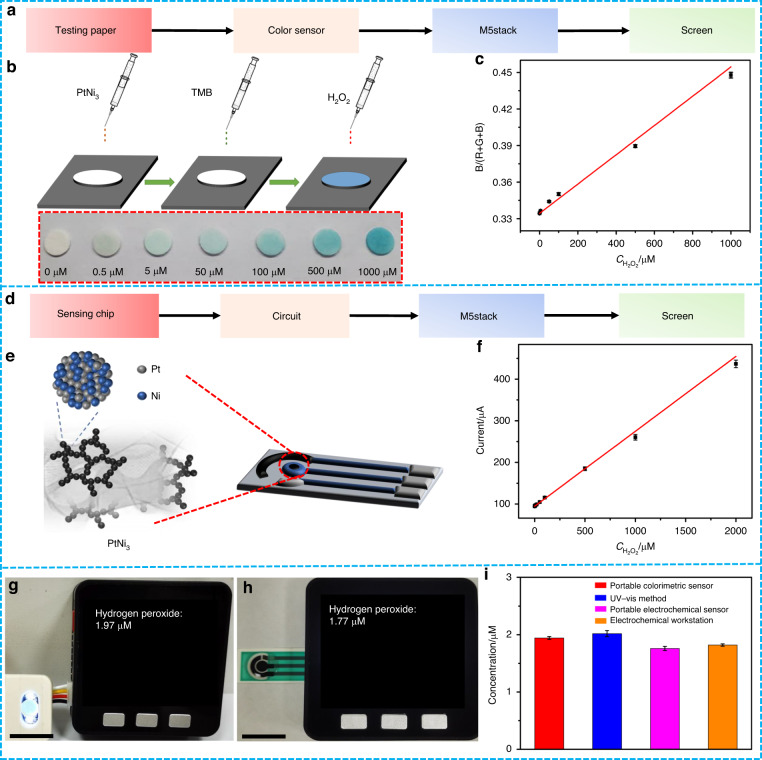
Diagram and application of the portable visual and electrochemical H_2_O_2_ sensors. **a**–**c** Schematic illustration and corresponding calibration curve of portable visual H_2_O_2_ sensing based on testing paper. **d**–**f** Schematic illustration and corresponding calibration curve of portable electrochemical H_2_O_2_ sensing. **g**, **h** Measurement of H_2_O_2_ released from HeLa cells with portable visual and electrochemical sensors. Scale bar = 1.0 cm. **i** Comparison of H_2_O_2_ concentrations measured with the portable colorimetric sensor, the UV‒vis spectrophotometer, the portable electrochemical sensor, and the electrochemical workstation, respectively

Developing miniaturized, portable and cost-saving H_2_O_2_ sensors will fulfill personalized health care needs, which has important practical significance and broad future application prospects in the future^42^. To take advantage of the colorimetric and electrochemical H_2_O_2_ sensing strategies based on the excellent peroxide-like and electrocatalytic activities of the PtNi_3_ dual hydrogel, we used it to construct dual portable visual and electrochemical sensors for H_2_O_2_. For the visual H_2_O_2_ sensor, we first produced colorimetric test paper by dropping PtNi_3_ hydrogel and TMB on filter paper (Fig. 6a, b). After integration with an M5stack development board and a color sensor, a portable visual H_2_O_2_ platform was successfully constructed. When the sample containing H_2_O_2_ was dropped onto the test paper, a color change was observed within 3 min. Then, this color signal was simultaneously captured and converted into R/G/B data by a color sensor. The microprocessor in an M5stack board can transform these data into the H_2_O_2_ concentration and directly display the information on a screen. The calibration curve between the H_2_O_2_ concentrations and the values of (B/(R + G + B)) at 25 °C was obtained as described in Table S3 and is shown in Fig. 6c, revealing excellent linearity in the concentration range of 0.50 µM–1.0 mM. It should be noted that the ambient temperature and humidity had little influence on the performance of the test paper (Figs. S10 and S11) due to its rapid response. Moreover, we designed a portable electrochemical H_2_O_2_ sensor with the aid of an in-house built signal processing circuit and an M5stack board, which were connected to the PtNi_3_ hydrogel-modified SPE (Fig. 6d, e). The current output of the SPE sensing chip was processed by the circuit and transmitted to the M5stack board. Then, the measured H_2_O_2_ concentrations were obtained according to the calibration curve (see Fig. 6f) (linearity range: 0.50 µM–2.0 mM) and synchronously displayed on the screen. With the advantages of high integration, simple operation, portable detection and low cost, the as-fabricated visual and electrochemical sensors show promise for convenient H_2_O_2_ determination without professional instruments and operators.

Considering the importance of H_2_O_2_ analysis in living cells, we used these two kinds of portable sensors to measure the H_2_O_2_ content in CHAPS-stimulated HeLa cells. For the colorimetric sensor, the measured H_2_O_2_ concentration was 1.97 µM (see Fig. 6g) within 3 min, which coincided well with of the result obtained using a UV‒vis spectrophotometer (2.08 µM) shown in Fig. 3f. For the electrochemical sensor, the content of H_2_O_2_ secreted from the HeLa cells was measured as 1.77 µM in 6 s, which was also in good agreement with the value determined by an electrochemical workstation (1.84 µM) (Fig. 6h, i). These results demonstrated the reliability of our H_2_O_2_ sensors in practical application. Moreover, to further verify the accuracy of the prepared sensors, we conducted comparative experiments in 0.1 PBS (pH 5.0) under different concentrations of H_2_O_2_ with the portable colorimetric sensor, the UV‒vis spectrophotometer, the portable electrochemical sensor, and the electrochemical workstation. The results shown in Table S4 indicated that the developed H_2_O_2_ sensors maintained high accuracy at different sample concentrations. In addition to the advantages of high integration and portable detection, electrochemical sensors can enable faster and more accurate H_2_O_2_ determination.

## Conclusions

In summary, this work demonstrated portable H_2_O_2_ sensors based on a multifunctional PtNi_3_ hydrogel, which exhibited superior peroxidase-like and electrocatalytic activities toward H_2_O_2_. The favorable catalytic properties of the 3D porous PtNi_3_ hydrogel benefited from the unique dual structures of alloyed Pt-Ni nanowires and moderate Ni(OH)_2_ nanosheets, which significantly enhanced the catalytic efficiency and the reaction affinity, respectively. Based on this, the colorimetric and electrochemical H_2_O_2_ sensing platforms displayed excellent analytic performances, such as wide linearity ranges (0.10 μM–10.0 mM & 0.50 μM–5.0 mM), low LODs (0.030 μM & 0.15 μM), and outstanding long-term stability for as long as 60 days. Ultimately, portable visual and electrochemical H_2_O_2_ sensors were successfully constructed and applied to the determination of H_2_O_2_ released from HeLa cells with the aid of sensing unit (test-paper and SPE) signal transformation (color sensor and signal processing circuit) and an M5stack development board. This work not only proves the great potential of versatile metallic hydrogels in H_2_O_2_ sensing but also introduces a new approach for the development of portable sensing devices in practical applications.

## Materials and methods

### Reagents and materials

Chloroplatinic acid hydrate (H_2_PtCl_6_·6H_2_O), nickel chloride hexahydrate (NiCl_2_·6H_2_O), NaBH_4_, H_2_O_2_, UA and AA were purchased from Sigma‒Aldrich. Glucose, TMB, sodium phosphate dibasic (Na_2_HPO_4_), sodium dihydrate phosphate anhydrous (NaH_2_PO_4_), and CHAPS were purchased from Aladdin. TA, KCl, NaCl, and hydrochloric acid (HCl) were purchased from Sinopharm. All the chemicals were of analytical grade, and all solutions were freshly prepared with ultrapure water (18.2 MΩ·cm). PBS, which was employed as the supporting electrolyte in the electrochemical experiments, was adjusted to different pH values by HCl, NaH_2_PO_4_ and Na_2_HPO_4_.

### Synthesis of the Pt-Ni hydrogels

A simple coreduction method was employed to prepare the Pt-Ni hydrogels^43^. Specifically, 46.80 μL of H_2_PtCl_6_ solution (205.0 mM) and 2.88 mL of NiCl_2_ solution (10.0 mM) were mixed with 39.50 mL of water and stirred for 10 min. Then, 0.050 mmol of NaBH_4_ was added under vigorous stirring, and the color of the solution turned immediately from light yellow to dark brown. After 2 min of continuous stirring followed by standing for 24 h in a dark room, a PtNi_3_ hydrogel with a Pt:Ni molar ratio of 1:3 was obtained. The prepared hydrogel was washed by removing and replacing the supernatant with deionized water 7 times. Finally, the concentration of the PtNi_3_ hydrogel was adjusted to 0.050 μg_(PtNi3)_ mL^−1^ and 1 mg_(PtNi3)_ mL^−1^ for the subsequent colorimetric and electrochemical experiments. The PtNi and PtNi_5_ hydrogels were synthesized by tuning the ratio of H_2_PtCl_6_ to NiCl_2_ accordingly. The molar mass of the added NaBH_4_ was also adjusted with the amounts of the precursors. Pure Pt and Ni hydrogels were obtained through the same route by using H_2_PtCl_6_ or NiCl_2_ as the only metal precursors.

### Construction of the dual portable H_2_O_2_ biosensors

The PtNi_3_-TMB solution was first prepared by mixing 10.0 μL of PtNi_3_ hydrogel (0.050 μg mL^−1^) and 10.0 μL of TMB (50.0 mM) into 1.0 mL of PBS (0.10 M, pH 5.0). Colorimetric test paper was fabricated by dropping 20.0 μL of PtNi_3_-TMB solution onto a round filter paper (*Φ* = 5.0 mm). Then, the test paper was fixed on a color sensor (30.0 mm◊23.0 mm◊8.0 mm), which was integrated with a TCS3472 chipset. The color sensor was operated by shining an LED through the test paper, and the reflected light was absorbed by a 3◊4 array of photodiodes (3 had red filters, 3 had green filters, 3 had blue filters and 3 had no filter). Then, the generated photocurrent was converted into an RGB-related digital signal by the analog-to-digital conversion module and transmitted to the M5stack development board (53.0 mm◊53.0 mm◊17.0 mm), which is a portable and highly integrated platform including an ESP32 system as well as a built-in battery module.

**Scheme 1:**
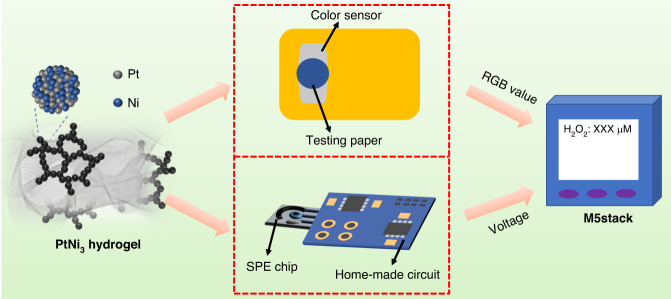
Schematic illustration of dual portable H_2_O_2_ biosensors based on the peroxidase-like and electrocatalytic activities of the PtNi_3_ hydrogel

For the portable electrochemical H_2_O_2_ sensor, 3.0 μL of PtNi_3_ hydrogel (1.0 mg mL^−1^) was modified on the SPE, in which a working electrode, a counter electrode and a reference electrode were integrated on a polyethylene glycol terephthalate (PET) film (30.0 mm◊9.0 mm◊0.20 mm) via the screen printing method. With the help of an in-house built signal processing circuit (30.0 mm◊25.0 mm◊1.50 mm) as well as the M5stack board, this portable sensor can be used to detect the current responses toward H_2_O_2_ from the PtNi_3_ hydrogel-modified SPE (illustrated in Scheme 1). Then, the amplified current responses were transformed to digital signals and transmitted to the M5stack development board.

In the experiments on measuring H_2_O_2_ released by living cells, 0.50 μM CHAPS was added to a solution with HeLa cells (3.60 × 10^5^ cells mL^−1^). Then, H_2_O_2_ was released from the stimulated HeLa cells and measured by the as-fabricated H_2_O_2_ sensors.

### Apparatus and measurement

SEM images were obtained from FEISEM (NANOSEM450, USA) upon an accelerating voltage operating at 15.0 kV. TEM was carried out on an FEI Talos S-FEG (Thermo Scientific, USA). XRD was conducted on a CRD-7000 (Shimadzu, Japan), and XPS was performed on a Model K-Alpha (Thermo Fisher Scientific Company, USA). The UV‒vis spectra data were recorded on a U-3900H spectrophotometer (Hitachi, Japan). Inductively coupled plasma‒optical emission spectrometry (ICP‒OES) was carried out on an ICAP7600DUO optical emission spectrometer (Spectro, Germany).

All electrochemical characterizations (i.e., CVs and i-t curves) were performed on a 660E electrochemical workstation (Shanghai Chenhua Instrument Corporation, China) with a single0compartment, three-electrode cell at room temperature. A Pt-Ni hydrogel-modified glassy carbon electrode (PtNi_x_/GCE) was used as the working electrode. A Ag/AgCl electrode and platinum wire were used as the reference electrode and the counter electrode, respectively.

### Supplementary information

Supporting Information
