# Supplementary material for: Portable visual and electrochemical detection of hydrogen peroxide release from living cells based on dual-functional Pt-Ni hydrogels
Source: Microsyst Nanoeng. 2023 Nov 29;9:152. doi: 10.1038/s41378-023-00623-y (PMC10684573; doi:10.1038/s41378-023-00623-y)
Supplement: Supplementary file 1 — Supporting Information [file 41378_2023_623_MOESM1_ESM.docx]

Supporting Information

Portable visual and electrochemical detection of hydrogen peroxide release from living cells based on dual-functional Pt-Ni hydrogels

Guanglei Li^1,2^, Yao Chen^1^, Fei Liu^1^, Wenhua Bi^1^, Chenxin Wang1^2^, Danfeng Lu^3^ and Dan Wen^1*^

Correspondence: Dan Wen ([dan.wen@nwpu.edu.cn](mailto:dan.wen@nwpu.edu.cn), ORCID: 0000-0001-6879-7982)

^1^State Key Laboratory of Solidification Processing, School of Materials Science and Engineering, Northwestern Polytechnical University (NPU) and Shaanxi Joint Laboratory of Graphene, Xi’an, 710072, P. R. China.

^2^Interdisciplinary Research Center of Biology & Catalysis, School of Life Sciences, NPU, Xi'an, 710072, P. R. China.

^3^Faculty of Printing, Packaging Engineering, and Digital Media Technology, Xi’an University of Technology, Xi’an, 710048, P. R. China.


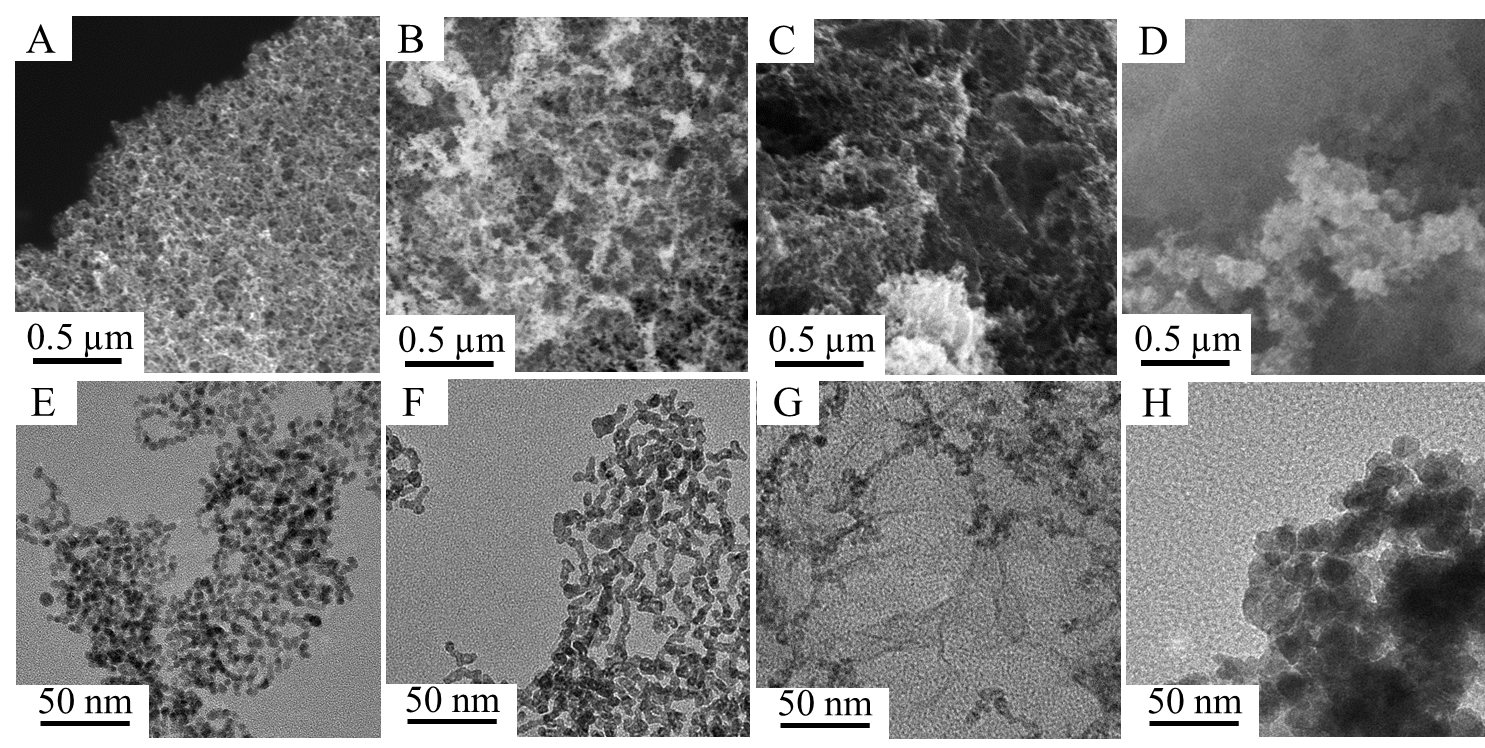


**Fig. S1** The scanning electron microscopy (SEM) image of the (A) pure Pt, (B) PtNi, (C) PtNi_5_ and (D) pure Ni hydrogels. The transmission electron microscopy (TEM) images of the (E) pure Pt, (F) PtNi, (G) PtNi_5_ and (H) pure Ni hydrogels.


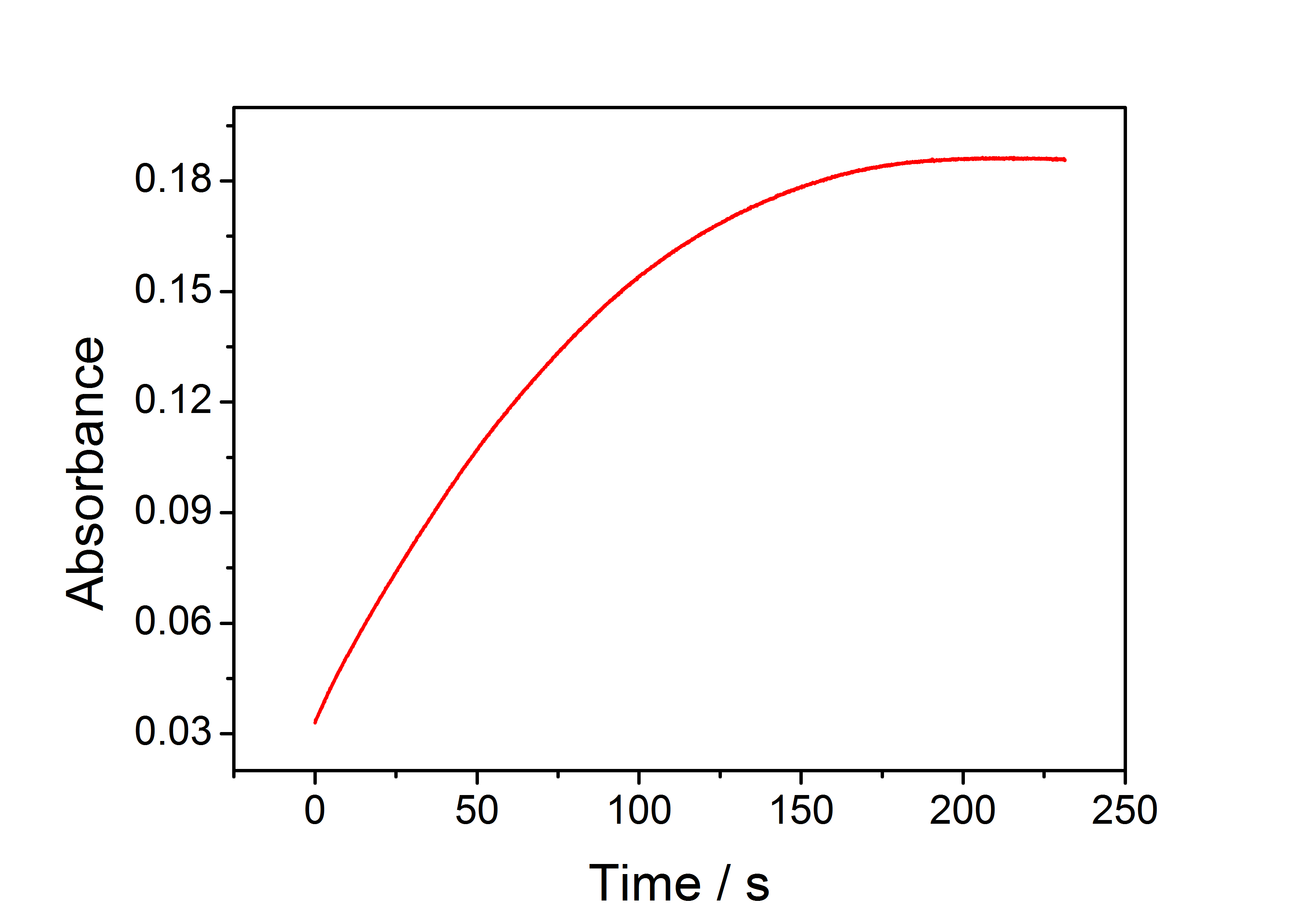


**Fig. S2** Time-dependent absorbance at 652 nm measured from the reaction solutions containing 0.5 mM hydrogen peroxide (H_2_O_2_), 0.5 mM 3,3,5,5-tetramethylbenzidine (TMB), and 0.05 μg mL^-1^ PtNi_3_ hydrogel in 0.1 M phosphate buffer solution (PBS) (pH 5.0) at room temperature.


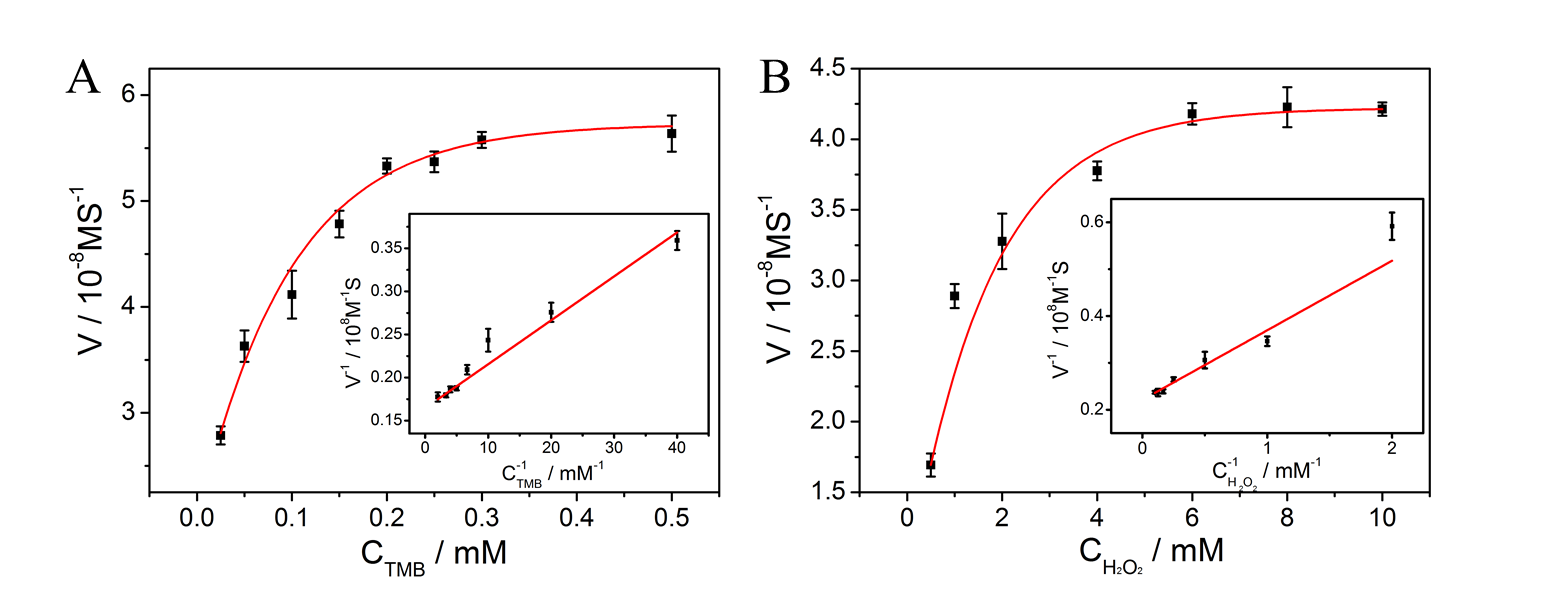


**Fig. S3** Lineweaver-Burk curves and double-reciprocal diagrams of the initial reaction rates of the PtNi_3_ hydrogel for the (A) TMB and (B) H_2_O_2_.


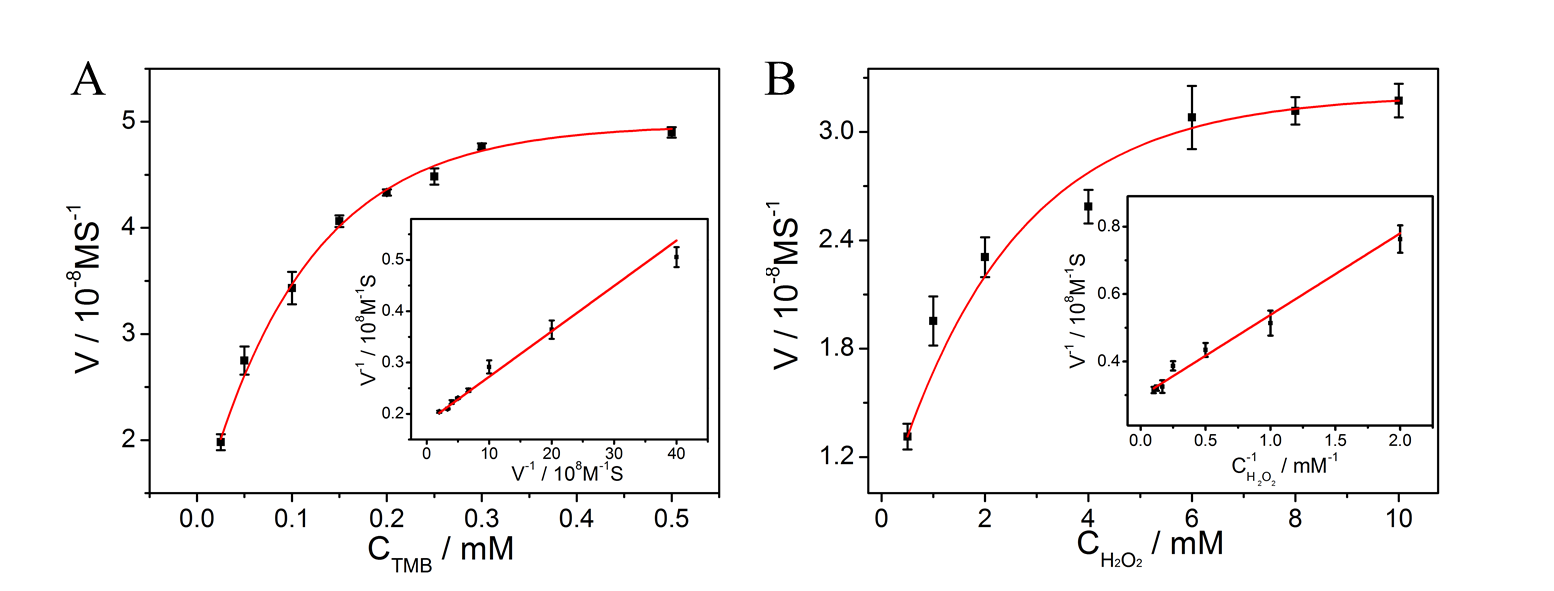


**Fig. S4** Lineweaver-Burk curves and double-reciprocal diagrams of the initial reaction rates of the PtNi hydrogel for the (A) TMB and (B) H_2_O_2_.


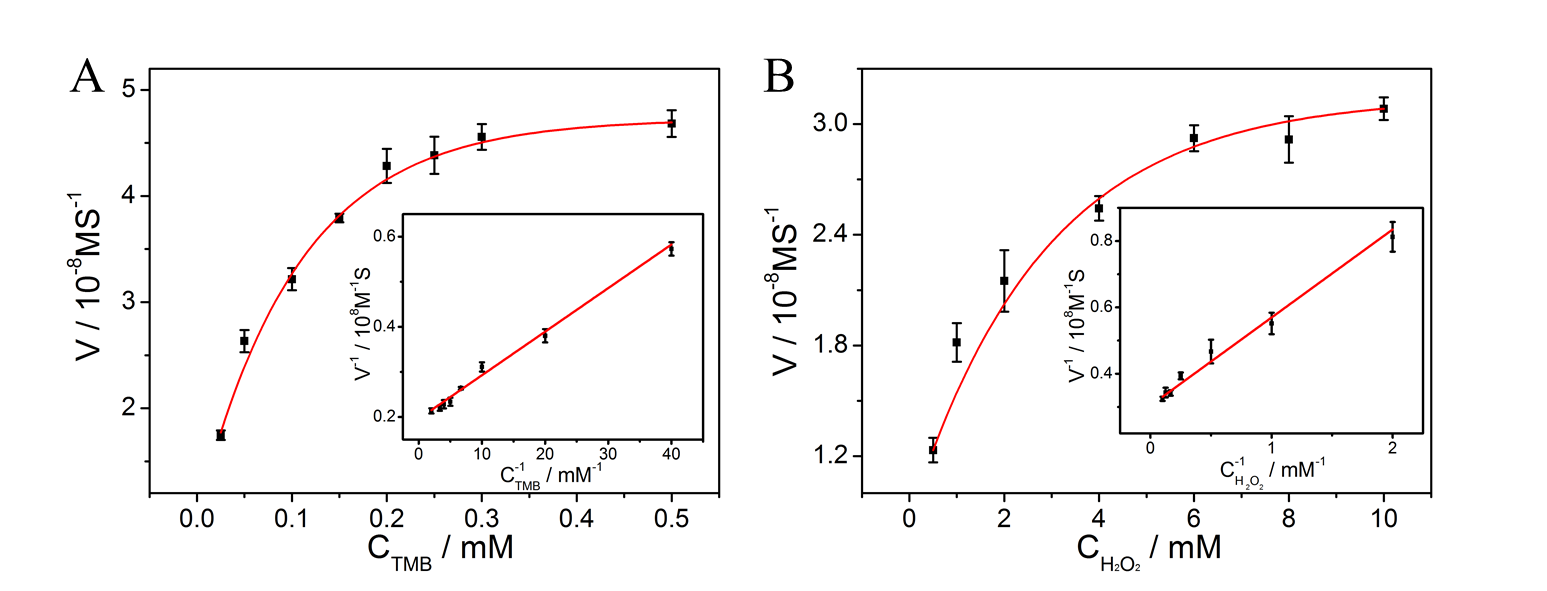


**Fig. S5** Lineweaver-Burk curves and double-reciprocal diagrams of the initial reaction rates of the PtNi_5_ hydrogel for the (A) TMB and (B) H_2_O_2_.


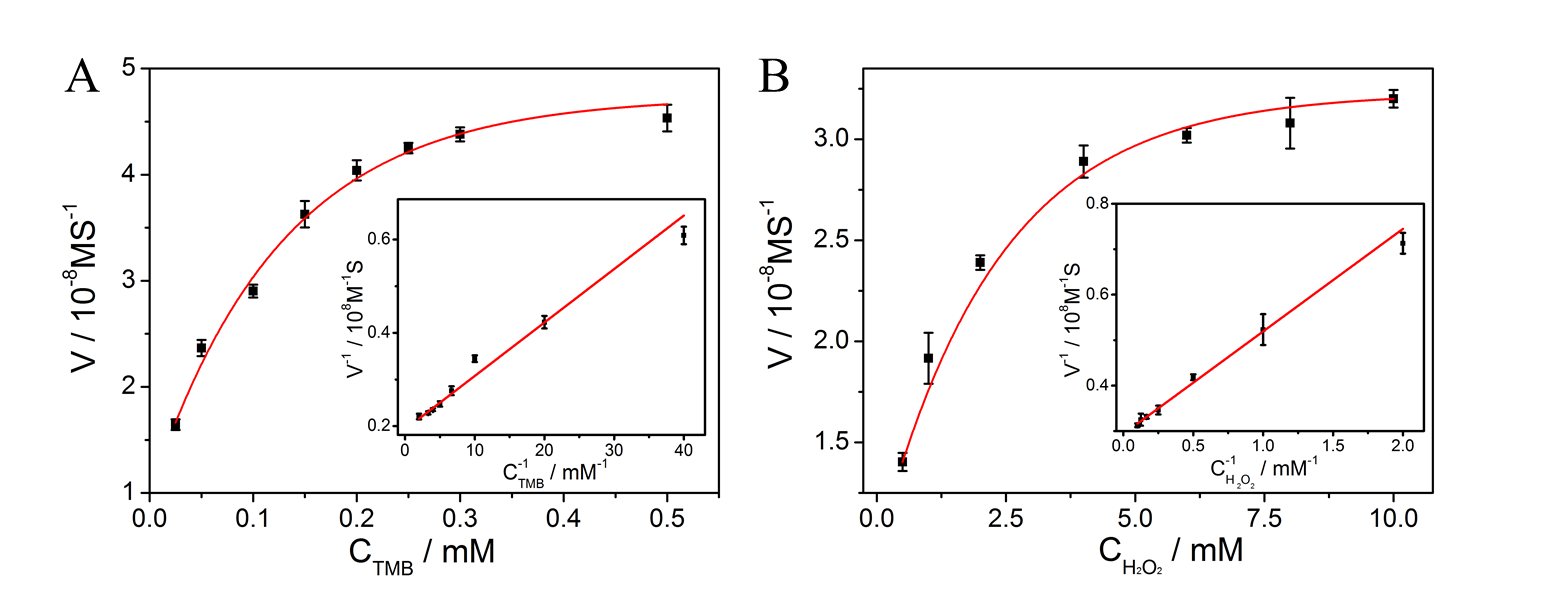


**Fig. S6** Lineweaver-Burk curves and double-reciprocal diagrams of the initial reaction rates of the Pt hydrogel for the (A) TMB and (B) H_2_O_2_.


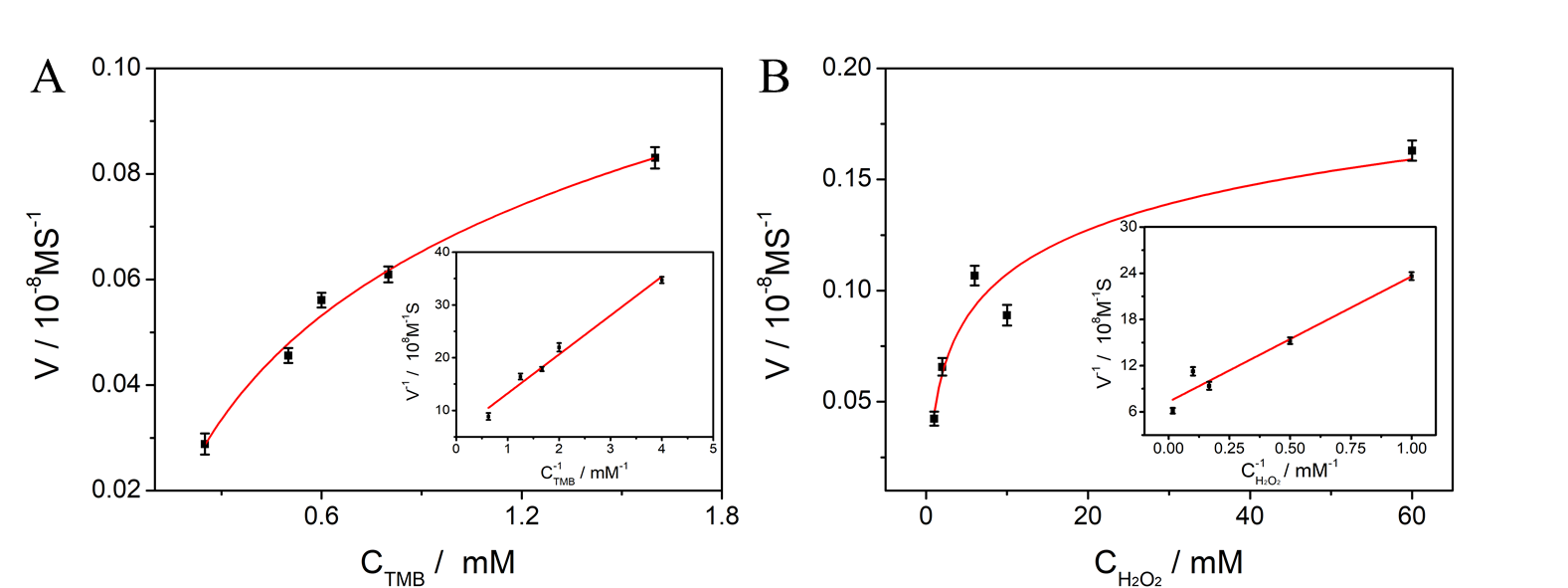


**Fig. S7** Lineweaver-Burk curves and double-reciprocal diagrams of the initial reaction rates of the Ni hydrogel for the (A) TMB and (B) H_2_O_2_.


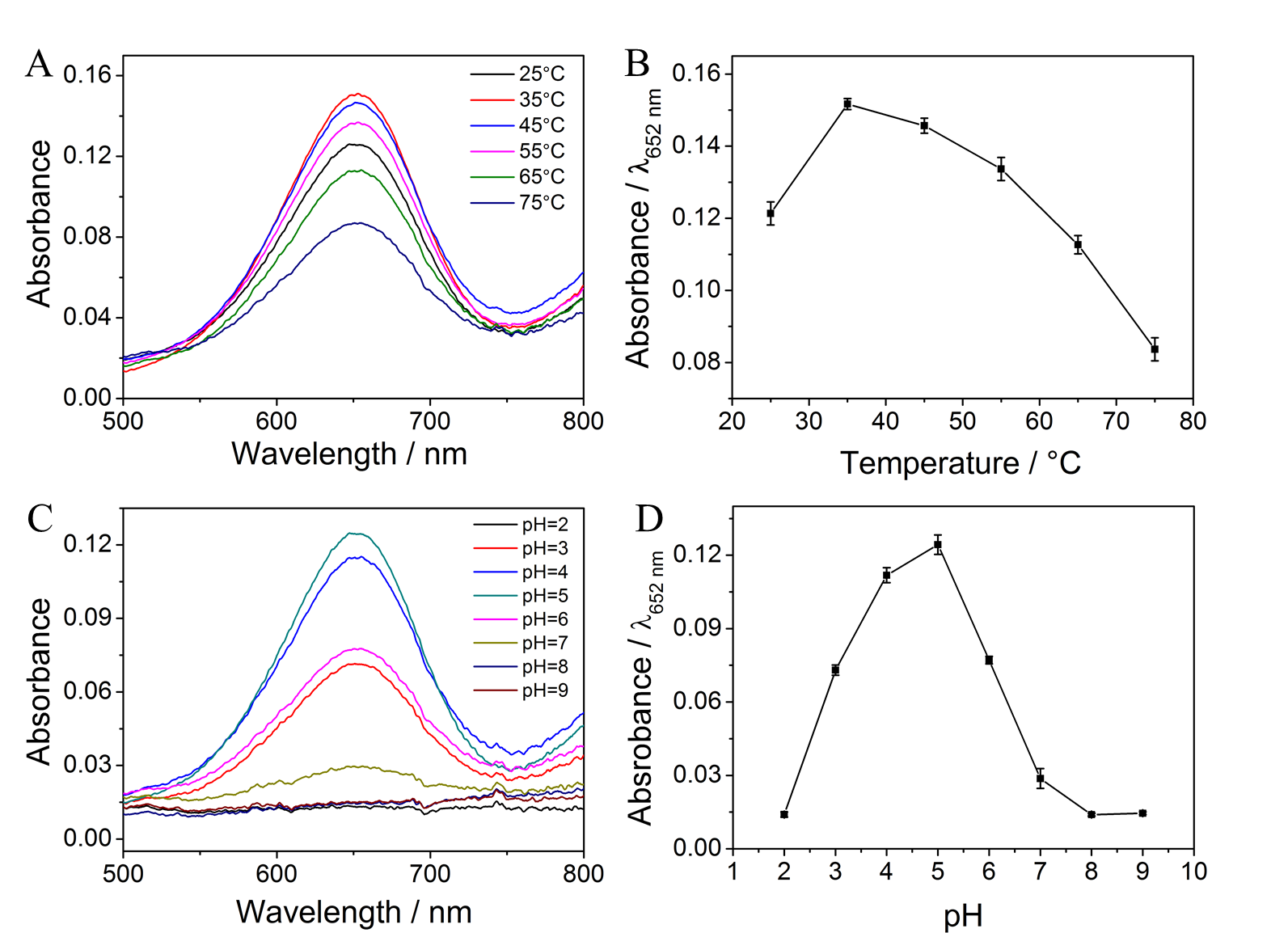


**Fig. S8** UV-vis absorption spectra of the PtNi_3_ hydrogel at (A-B) a temperature gradient of 25-75 ℃ and (C-D) a pH range of 2-9 in the presence of 0.5 mM TMB and 0.2 mM H_2_O_2_.


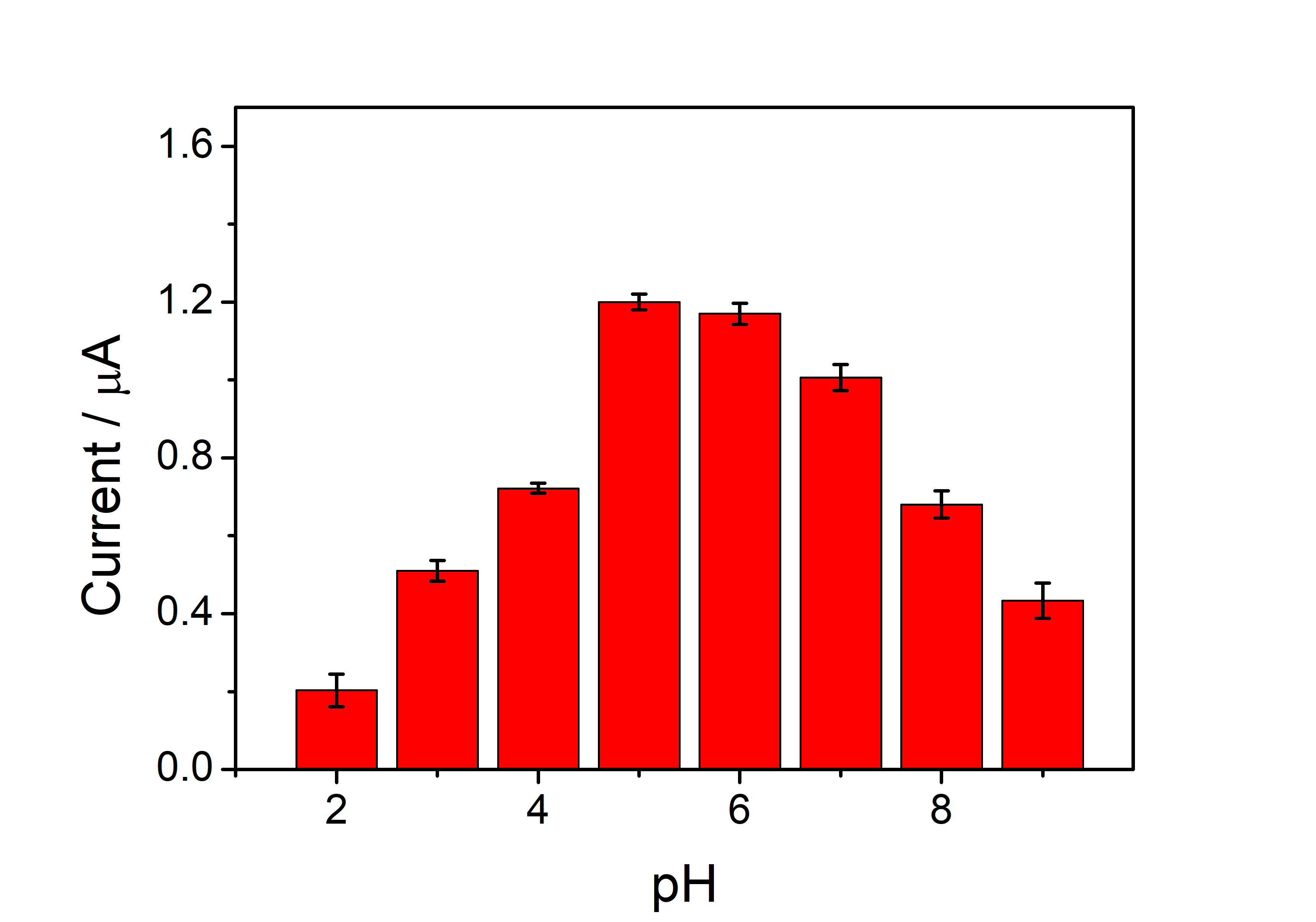


**Fig. S9** The influence of pH on the electrocatalytic activity of the PtNi_3_ hydrogel.


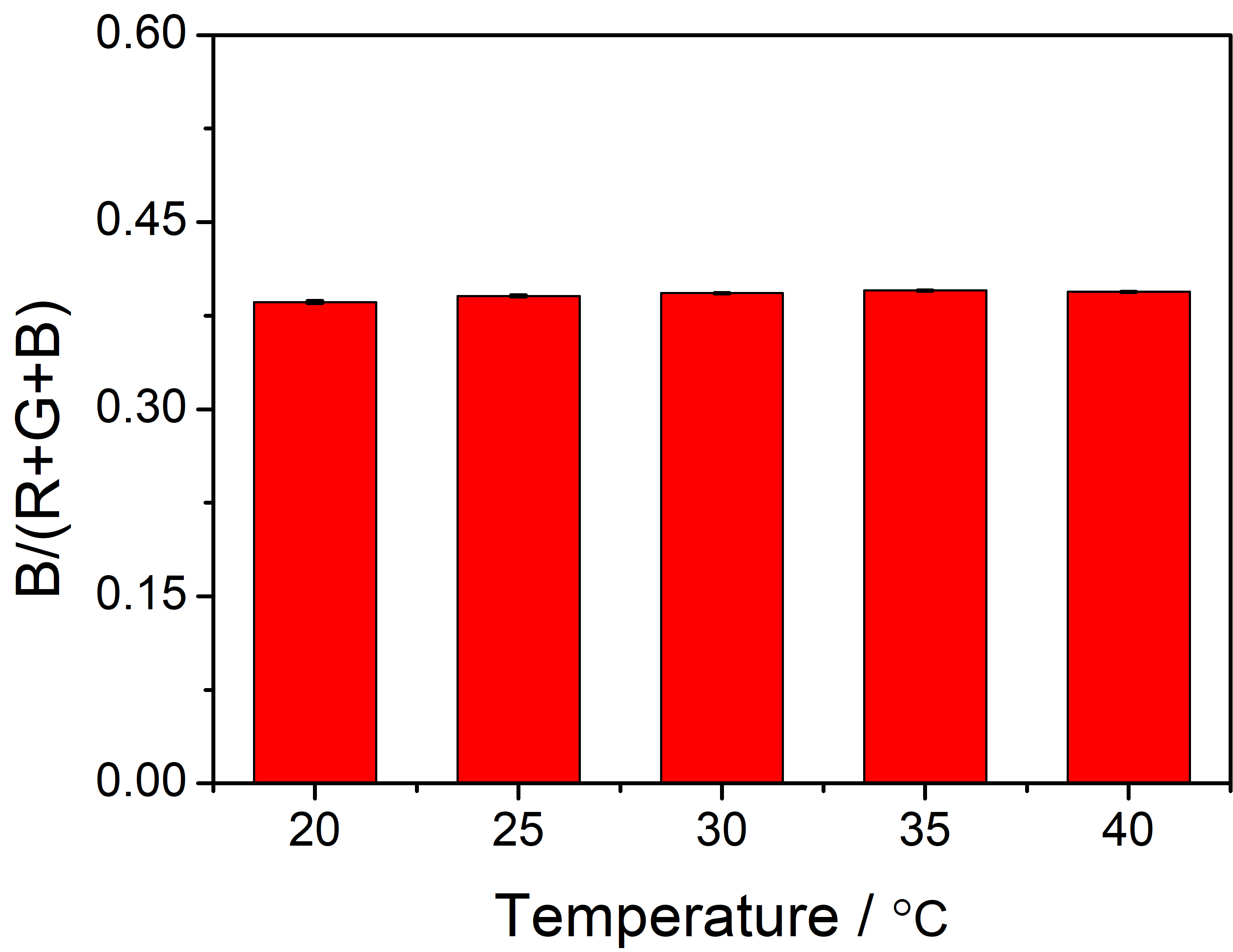


**Fig. S10** The influence of ambient temperature on the colorimetric test-paper.


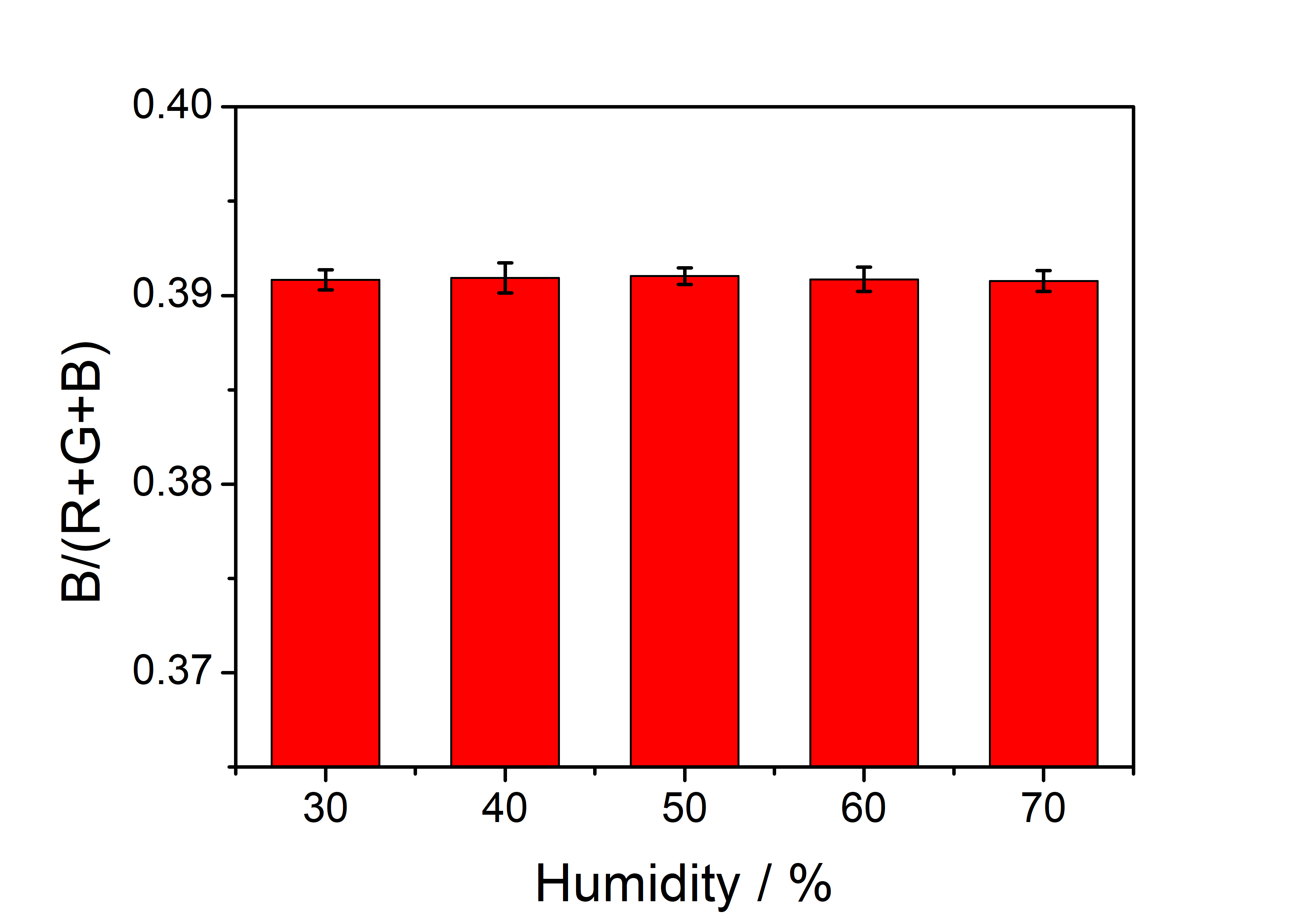


**Fig. S11** The influence of ambient humidity on the colorimetric test-paper.

**Table S1:** Comparison of the kinetic parameters of the PtNi_x_, pure Pt, Ni hydrogels and other artificial/natural enzymes.

| Samples | *K_m_* (mM) | | *V_max_* (10^-8^ Ms^-1^) | | *K_cat_* (s^-1^) | | Ref. |
| --- | --- | --- | --- | --- | --- | --- | --- |
|  | TMB | H_2_O_2_ | TMB | H_2_O_2_ | TMB | H_2_O_2_ |  |
| PtNi_3_ hydrogel | 0.031 | 0.67 | 6.08 | 4.52 | 4.50×10^4^ | 3.35×10^4^ | This work |
| PtNi hydrogel | 0.048 | 0.818 | 5.43 | 3.37 | 2.76×10^4^ | 1.71×10^4^ | This work |
| PtNi_5_ hydrogel | 0.049 | 0.873 | 5.10 | 3.28 | 2.37×10^4^ | 1.29×10^4^ | This work |
| Pt hydrogel | 0.059 | 0.904 | 5.18 | 3.40 | 2.54×10^4^ | 1.87×10^4^ | This work |
| Ni hydrogel | 1.26 | 2.23 | 0.17 | 0.14 | 2.02×10^3^ | 1.62×10^3^ | This work |
| Pd@Pt core-frame | 0.43 | 14 | 5.10 | 9.0 | 7.20×10^-3^ | 1.30×10^-2^ | 1 |
| Pt/hBNNSs | 0.21 | 9.2 | / | / | / | / | 2 |
| Pt nanodendrites | 0.81 | 6.90 | 12 | 9.90 | 1.70×10^-2^ | 1.40×10^-2^ | 1 |
| Fe-N-C SAzymes | 3.6 | 12 | 116 | 35.6 | 0.78 | 0.24 | 3 |
| Fe-AL-E | 20.10 | 58.0 | 5.43 | 1.12 | 8.83×10^-5^ | 1.82×10^-5^ | 4 |
| Pd cubes | 0.054 | 700 | 9.7 | 6.5 | 6.9×10^4^ | 4.6×10^4^ | 5 |
| Mo_SA_-N_3_-C | 0.79 | / | / | 37 | 4.0×10^4^ | / | 6 |
| HRP | 0.43 | 3.70 | 10.0 | 8.71 | 4.0×10^3^ | 3.48×10^3^ | 7 |

**Table S2:** The comparison of the sensing performances between the as-prepared colorimetric H_2_O_2_ sensor and the others recently reported in the literatures.

| Sensing material | Linearity  range | Limit of detection | Long-term stability (signal retention) | Ref. |
| --- | --- | --- | --- | --- |
| PtNi_(1:3)_ hydrogel | 0.1 μM-10 mM | 0.03 μM | 60 days | This work |
| Pt nanoclusters | 0-200 μM | 0.46 μM | 1 months | 8 |
| B, N-PdRu aerogel | 10 μM-2 mM | 6 μM | / | 9 |
| Fe-AL | 10-100 mM | 54 μM | / | 10 |
| Fe_0.8_Ni_0.2_S_2_ | 1.25-12.5 μM | 0.77 μM | / | 11 |
| MSA/Cu NCs | 1 μM-1 mM | 0.5 μM | 10 months | 12 |
| PtNPs/Cu-TCPP(Fe) | 2-100 μM | 0.357 μM | / | 13 |
| Fe/CuSn(OH)_6_ | 30-1000 μM | 9.49 μM | 11 days | 14 |
| cysteine/I^-^/Au NPs | 0-80 μM | 2 μM | / | 15 |

**Table S3:** The measured R, G, B values at different concentrations of H_2_O_2_.

| H_2_O_2_ (μM) | B/(R+G+B) |
| --- | --- |
| 0 | 0.33433 |
| 0.5 | 0.33448 |
| 5 | 0.33645 |
| 50 | 0.34439 |
| 100 | 0.35086 |
| 500 | 0.39023 |
| 1000 | 0.44915 |

**Table S4.** The comparison of the H_2_O_2_ concentrations measured with the portable colorimetric sensor, the UV-vis spectrophotometer, the portable electrochemical sensor, and the electrochemical workstation, respectively.

| Setting concentration | Portable colorimetric sensor | UV-vis spectrophotometer | Portable electrochemical sensor | Electrochemical workstation |
| --- | --- | --- | --- | --- |
| 10 µM | 9.31 µM | 9.90 µM | 9.58 µM | 10.04 µM |
| 50 µM | 46.27 µM | 49.54 µM | 47.78 µM | 49.88 µM |
| 100 µM | 95.37 µM | 101.21 µM | 96.54 µM | 99.52 µM |
| 500 µM | 481.61 µM | 493.70 µM | 489.16 µM | 502.5 µM |

**Supplemental** **References**

1. Wu, R. F. et al. *Adv. Funct. Mater*. **28**, 1801484 (2018).
2. Ivanova, M. N. et al. *ACS Appl. Mater. Inter.* **11**, 22102-22112 (2019).
3. Jiao, L. et al. *Anal. Chem*. **91**, 11994-11999 (2019).
4. Li, L. J. et al. *ACS Sustain. Chem. Eng.* **9**, 12833-12843 (2021).
5. Xia, X. H. et al. *ACS Nano* **9**, 9994-10004 (2015).
6. Wang, Y. et al. *Chem* **7**, 436-449 (2021).
7. Gao, L. et al. *Nat. Nanotechnol*. **22**, 577-583 (2007).
8. Jin, L. H. et al. *ACS Appl. Mater. Inter.* **9**, 10027-10033 (2017).
9. Zeng, Y. T. et al. *ACS Appl. Mater. Inter*. **13**, 36816-36823 (2021).
10. Li, L. J. et al. *ACS Sustain. Chem. Eng*. **9**, 12833-12843 (2021).
11. Zhang, C. Y. & Nan, Z. D. *J. Phys. Chem. C* **126**, 4355-4364 (2022).
12. Du, Y. B., Fang, J., Wang, H. L. & Yang, Y. *ACS Appl. Mater. Inter.***9**, 11035-11044 (2017).
13. Chen, H. Y. et al. ACS *Appl. Mater. Inter.* **10**, 24108-24115 (2018).
14. Liu, H. et al. *ACS Sustainable Chem. Eng*. **6**, 14383-14393 (2018).
15. Wang, F., Liu, X. Q., Lu, C. H. & Willner, I. *ACS Nano* **7**, 7278-7286 (2013).
